# ITCH-Mediated Ubiquitylation of ITGB3 Promotes Cell Proliferation and Invasion of Ectopic Endometrial Stromal Cells in Ovarian Endometriosis

**DOI:** 10.3390/biomedicines11092506

**Published:** 2023-09-11

**Authors:** Liansuo Zhang, Wei Shao, Mingqing Li, Songping Liu

**Affiliations:** 1 Department of Obstetrics and Gynecology, Jinshan Hospital, Fudan University, Shanghai 201508, China; ls_zhang@fudan.edu.cn (L.Z.); shaowei@fudan.edu.cn (W.S.); 2 Laboratory for Reproductive Immunology, Hospital of Obstetrics and Gynecology, Fudan University, Shanghai 200080, China; 3 Shanghai Key Laboratory of Female Reproductive Endocrine Related Diseases, Hospital of Obstetrics and Gynecology, Fudan University, Shanghai 200080, China

**Keywords:** endometriosis, ubiquitination, ITGB3, ITCH

## Abstract

Post-translational modification of proteins is involved in the occurrence of endometriosis (EM); however, the role of ubiquitination modification in EM remains unclear. Integrin β3 (ITGB3) is one of the β-subunits of integrins, which plays a key role in tumor progression. In this study, we investigated the roles of ITGB3 and ITCH, one of the ubiquitin E3 ligases, in ectopic endometrial stromal cells (ESCs) and EM. Primary ectopic ESCs and normal ESCs were isolated and purified. Western blot was used to detect the expression of ITGB3 and ITCH in ESCs. The interaction between ITGB3 and ITCH in ESCs was investigated by the co-immunoprecipitation and ubiquitylation analysis. With or without the overexpression of ITCH and/or ITGB3, the proliferation and invasion of ectopic ESCs were detected by the CCK8 assay and transwell migration assay, respectively. We found that ITGB3 is upregulated in ectopic ESCs from patients with EM. ITCH interacts with ITGB3 by co-immunoprecipitation, and ITCH-overexpressing significantly increased the ubiquitination of ITGB3. The data of the CCK8 assays showed that ITGB3 overexpression significantly promoted cell proliferation of ectopic ESCs at 12, 24, 48, and 72 h. The transwell migration assays showed that ITGB3 overexpression significantly enhanced the invasive ability. However, ITCH had the opposite effects in both assays. Our findings indicate that ITCH-mediated ubiquitylation of ITGB3 regulates the proliferation and invasion ability of ectopic ESCs in EM.

## 1. Introduction

EM is a common estrogen-dependent chronic disease characterized by the presence and growth of endometrial-like glands and stroma at sites outside the uterus [1]. The main manifestations of EM include dyspareunia, abnormal menstruation, pelvic mass, pelvic pain, and infertility, which affect nearly 10% of women of childbearing age [2]. Related symptoms of EM gradually weaken the woman’s ability to carry out some daily activities, which results in a perception of a worsening health condition and overall well-being [3]. At present, the continuously decreasing fertility rate is gradually attracting the attention of researchers. Upon exploring the causes, EM is found to be a crucial factor, requiring even an oocyte-freezing procedure for assisted reproductive pregnancy in the couple [4]. Of course, psychological factors are also important for the success of pregnancy in the progression of EM [5]. EM shares some malignant biological behaviors with cancer, such as hyperplasia, aggressive migration, and invasion, although it is generally considered a benign disease [6]. Similarly, ectopic ESCs from EM demonstrated increased proliferation, migration, and invasion compared with normal ESCs from non-endometriotic controls [7]. Currently, research on ectopic ESCs is a common way to explore the disease mechanism of EM. However, the precise etiology and pathogenesis of the disease remain controversial despite extensive research.

Integrins are dominant glycoproteins in many cell adhesion cascades. They are transmembrane glycoprotein receptors composed of non-covalently bound α and β subunits [8]. ITGB3, the gene encoding the extracellular matrix receptor β3 integrin, is one of the β-subunits of integrins that are established key players in the interaction between cells and their environment. ITGB3 plays a variety of important roles in tumor progression and reprogramming, which can affect angiogenesis through interaction with the microenvironment or small molecules [9]. In addition, ITGB3 interacts directly with other genes to affect cell migration and is regulated by non-coding RNA to affect cell migration [10]. Research shows that the expression of ITGB3 in breast cancer tissue and serum is increased, and silencing ITGB3 can inhibit the metastasis of breast cancer breast epithelial cells [11,12]. In our previous study, it was found that downregulation of lncRNA-H19 could inhibit ectopic endometrial cell proliferation and invasion by modulating miR-124-3p and ITGB3 expression [13], indicating that ITGB3 was possibly involved in the occurrence of EM.

Ubiquitination is one of the most important post-translational modifications, which plays a significant role in regulating the stability, activity, and location of cellular proteins. Ubiquitination governs protein degradation by utilizing the ubiquitin–proteasome system (UPS), including ubiquitin-activating enzyme (E1), ubiquitin-conjugating enzyme (E2), and ubiquitin–protein ligase (E3) [14]. E3 ubiquitin ligases are regarded as the most influential ubiquitin enzymes owing to their ability to select, bind, and recruit target substrates for ubiquitination, which determines the specificity of the ubiquitination system [15]. In recent years, the E3 ubiquitin ligases have been characterized to participate in developing various diseases, including EM [16,17]. However, it is still unclear whether ITGB3 can be modified by ubiquitination to participate in the development of EM. Bioinformatics analysis was performed using UbiBrowser_v1.0 (http://ubibrowser.bio-it.cn/ubibrowser/) (accessed on 30 June 2022) to identify the E3 ubiquitin ligases that can modify ITGB3. As a result, ITCH, a highly conserved E3 ubiquitin ligase, was found because of its higher confidence level.

Therefore, the current study aims to investigate the role and regulatory mechanism of ITGB3 and ITCH in the proliferation and invasion of ectopic ESCs in vitro.

## 2. Materials and Methods

### 2.1. Tissue Collection and Cell Culture

The ectopic EM lesions (located on the ovary) were obtained from women with EM (*n* = 10, age ranging from 25 to 44 years old, mean age 35.4 years old, III–IV according to the revised American Fertility Society classification) who underwent laparoscopic treatment in Jinshan Hospital affiliated with Fudan University. Normal endometrium, served as a control group, was obtained from women with uterine leiomyoma (*n* = 10, age ranging from 27 to 45 years old, mean age 35.1 years old). The clinical characteristics of the patients from the experimental group and control group were not significantly different. The study was approved by the Institutional Ethics Committee of Jinshan Hospital, affiliated with Fudan University, and written consent was provided and confirmed by all participants (NO. JIEC 2022-S16). Primary ESCs derived from ectopic endometria of women with EM or normal endometria of women without EM were isolated and cultured as follows: the collected endometrial tissues were washed with phosphate-buffered saline (PBS) and minced, then digested by 4% collagenase at 37 °C for 60 min; the dissociated tissues were sequentially filtered through a 70 µm and a 20 µm nylon mesh to remove epithelial cells; cell suspensions were further centrifuged, and then the cell deposition was resuspended in DMEM/F12 (Thermo, Saint Louis, MO, USA, 11,320,033) containing 10% fetal bovine serum (FBS, Gibco, Grand Island, NY, USA, 10,091,148) and 1% penicillin–streptomycin (Gibco, Grand Island, NY, USA, 15,070,063); and the cells were then cultured in an incubator with 5% CO_2_ at 37 °C. The medium was changed in the first 24 h and every 2–3 days following. The positive expression of Vimentin and negative expression of CK19 in ESCs were determined by immunocytochemistry.

### 2.2. Immunocytochemistry

Human ectopic or normal ESCs were seeded in a 6-well plate with coverslips, which were washed three times with PBS and then fixed with 4% paraformaldehyde for 30 min. Then, the cells were immersed in 3% H_2_O_2_ for 10 min to quench endogenous peroxidase. After being blocked with 1% bovine serum albumin (BSA) for 1 h, the cells above were incubated with primary antibodies of Vimentin and CK19 overnight at 4 °C and then were incubated with secondary antibodies for 0.5 h at room temperature. Finally, ESCs were stained with diaminobenzidine (DAB) and observed by microscope.

### 2.3. Western Blot

Western blot was adopted to detect protein expression levels of ITGB3, ITCH, and ubiquitin (Ub). Ectopic or normal ESCs were lysed using RIPA lysis buffer (Beyotime Biotechnology, Shanghai, China, P0013B) with protease inhibitor PMSF (Beyotime Biotechnology, Shanghai, China, ST506) and centrifugated at 1000× *g* at 4 °C. A micro-BCA protein assay kit (Servicebio, Wuhan, China, G2026) was used to determine protein concentrations. Proteins in cell lysates were transferred onto Polyvinylidene Fluoride (PVDF) membrane (Millipore, Bedford, MA, USA, IPVH00010) after being separated with 10% SDS-PAGE. Subsequently, they were blocked and incubated with primary antibodies for 12 h. After washing with TBST buffer, the membranes were incubated for 1 h at 37 °C, with secondary antibodies conjugated to horseradish peroxidase (1:1000, Beyotime Biotechnology, Shanghai, China). An enhanced chemiluminescence detection kit (Thermo, Saint Louis, MO, USA, 32,209) was used for visualization and quantification. Detailed antibody information is shown in Table 1.

### 2.4. RNA Isolation and Quantitative Real-Time PCR (qRT-PCR)

Total RNA was isolated from ectopic ESCs with TRIzol (Solarbio, Beijing, China, R1100) and then reversed into cDNA with a RevertAid First Strand cDNA Synthesis Kit (Thermo, Saint Louis, MO, USA, K1622) according to the manufacturer’s protocol. The mRNA expression was assessed using Applied BiosystEM Prism 7300 sequence detection system with Maxima SYBR Green/ROX qPCR Master Mix according to the manual. The 2^−ΔΔCt^ cycle threshold method was used to assess the relative quantification. Glyceraldehyde-3-phosphate dehydrogenase (GAPDH) was included as the internal control, and the primers used in this study are shown in Table 2.

### 2.5. Cell Transfection

ITGB3 overexpression (ITGB3-OE) plasmids, ITCH overexpression (ITCH-OE) plasmids, ITCH overexpression, and ITGB3 overexpression combined (ITCH-OE + ITGB3-OE) plasmids and control plasmids were purchased from Shanghai Yuanke Biotechnology Co., Ltd. (Shanghai, China, Y10026). Plasmids were transfected into ectopic ESCs using Lipofectamine 6000 (Beyotime Biotechnology, Shanghai, China, C0526) according to the manufacturer’s instructions. Then, the transfection efficiency was confirmed using Western blot and qRT-PCR.

### 2.6. Co-Immunoprecipitation and Ubiquitination Analysis

Cell lysates were homogenized using RIPA lysis buffer and then incubated with or without anti-ITCH antibody, anti-ITGB3 antibody, or control IgG overnight at 4 °C to confirm the interaction between ITCH and ITGB3. Next, an incubation with protein A/G Plus agarose beads (Beyotime Biotechnology, Shanghai, China, P2019) for 2 h at 4 °C was necessary. After being washed three times in lysis buffer, the immunoprecipitated complexes were analyzed by Western blotting. After being infected with ITCH-OE plasmids, the ectopic ESCs were lysed with RIPA buffer, and the lysates were reacted with anti-ITGB3 or control IgG antibodies, respectively. Finally, the immunoprecipitated complexes were analyzed with Western blotting using anti-ubiquitin.

### 2.7. Cell Proliferation Analysis

Cells were trypsinized and counted under a microscope, and cells were seeded into wells at a density of 3 × 10^3^ cells/well. Each 100 hundred μL of suspension was seeded in 96-well plates and cultured at 37 °C overnight. Ectopic ESCs were infected with ITCH-OE plasmids only, ITGB3-OE plasmids only, or both of them. After the completion of the culture at 0, 12, 24, 48, and 72 h, the cells were then incubated with 100 uL of DMEM/F12 medium containing 10 μL of Cell Counting Kit-8 (CCK-8, BBI Life Sciences, Shanghai, China, E606335) reagent at 37 °C for 1 h. Thereafter the absorbance value (optical density) at 450 nm, which indicates cell proliferation, was measured using a microplate reader.

### 2.8. Transwell Migration Assay

After the cells were transfected with ITCH-OE plasmids only, ITGB3-OE plasmids only, or both for 48 h, they were cultured in serum-free medium for 24 h. After adding 80 μL of Matrigel (BD Biocoat, Corning, NY, USA, 356,234), ectopic ESCs (3 × 10^5^/mL) were seeded to each insert (0.3 mL). In the lower portion of the 24-well plate, 0.7 mL of complete culture medium containing 10% FBS was added, and each group was cultured in a 37 °C incubator for 48 h. The inserts were fixed with 4% paraformaldehyde, stained with 0.1% crystal violet solution, and washed three times with PBS. A cotton swab was then used to carefully wipe away cells that did not migrate through the transwell chamber. The invading cells were counted under a microscope by magnification.

### 2.9. Statistical Analysis

All experiments were performed in triplicate. The results are expressed as mean ± standard deviations (SD). All statistics were analyzed by GraphPad Prism software Version 7.0 (GraphPad Software, Inc., La Jolla, CA, USA). Comparison between different experimental groups was performed with Student’s *t*-test or ANOVA. *p* < 0.05 was set as the significance level.

## 3. Results

### 3.1. Immunostaining Identification of Ectopic ESCs and Normal ESCs

**Figure 1:**
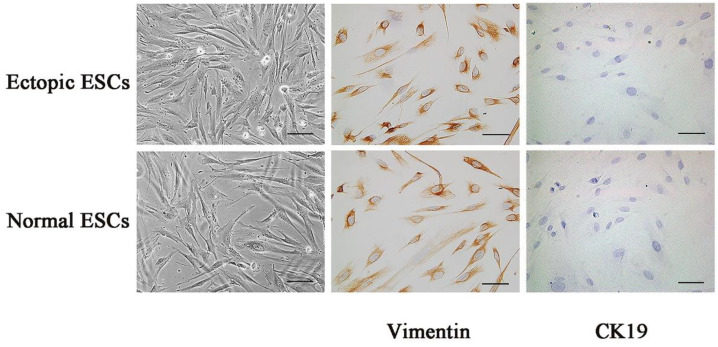
Immunostaining identification of ectopic ESCs and normal ESCs. Intracellular expression of cytoskeleton proteins Vimentin and CK19 was measured with immunohistochemistry in ectopic ESCs and normal ESCs. It was indicated that intracellular expression of Vimentin was positive, whereas that of CK19 was negative. The images of representative morphology are shown. Scale bar: 25 μm.

As shown in Figure 1, the immunostaining data of ectopic ESCs and normal ESCs showed that primary isolated endometrial stromal cells are negative for Cytokeratin 19(CK19), a cytokeratin specific for epithelial cells, but positive for Vimentin, a skeletal protein specific for stromal cells, suggesting the cell purity of Vimentin^+^ ESCs was beyond 95%.

### 3.2. ITGB3 Is Upregulated in Ectopic ESCs from Patients with EM

**Figure 2:**
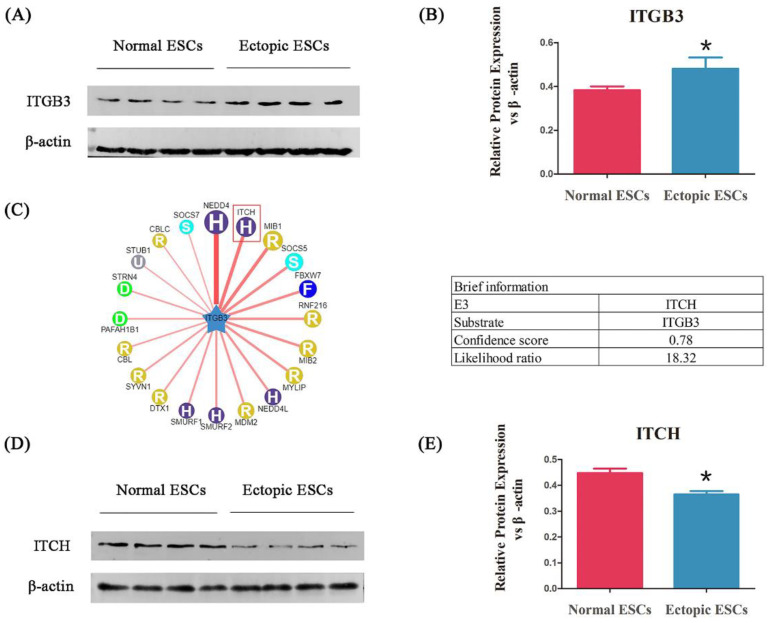
ITGB3 is upregulated in ectopic ESCs from patients with EM. Western blot analyses detecting ITGB3 relative expression levels (**A**). ITGB3 relative expression levels in the normal ESCs and the ectopic ESCs, * *p* < 0.05, compared to the normal group (**B**). Predicated human E3 ubiquitin ligases interact with ITGB3 and are shown in UbiBrowser alongside the brief information of ITCH (**C**). ITCH relative protein expression levels in the normal ESCs and the ectopic ESCs (**D**); * *p* < 0.05 compared with the normal group (**E**). Data are expressed as mean ± SD, and data between two groups were compared by two-tailed Student’s *t*-test.

To identify whether ITGB3 is involved in EM, the ESCs were collected from patients with EM and healthy controls, and the ITGB3 protein expression was measured by Western blotting. As shown in Figure 2A,B, ITGB3 protein expression was significantly increased in ectopic ESCs from patients with EM compared with normal ESCs from healthy controls. To further study the regulation of ITGB3 in EM, Bioinformatics analysis was performed using UbiBrowser_v1.0 (http://ubibrowser.bio-it.cn/ubibrowser/) (accessed on 30 June 2022) (Figure 2C) to find predicted E3 ubiquitin ligases. With a higher confidence level, ITCH was selected for further study in EM. Figure 2D,E show that ITCH protein levels were significantly downregulated in ectopic ESCs compared with normal ESCs. Thus, we hypothesized that ITGB3 may be associated with EM through interacting with ITCH.

### 3.3. ITCH Interacts with ITGB3 via Ubiquitination

**Figure 3:**
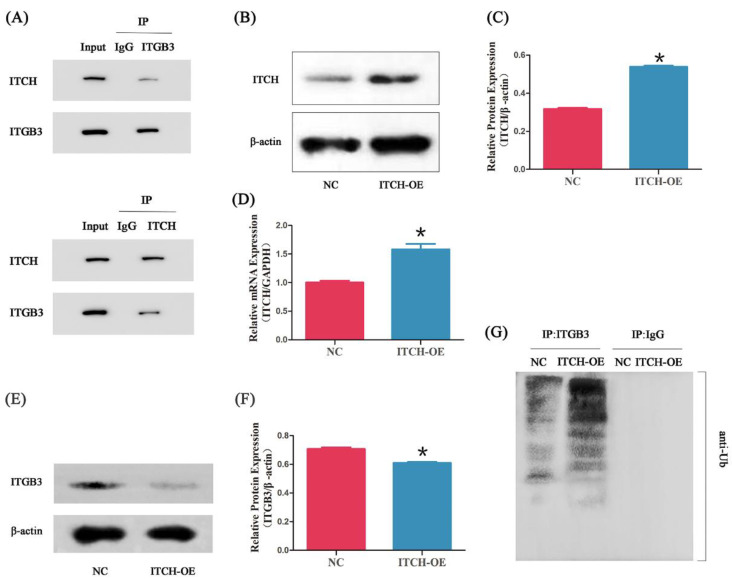
ITCH interacts with ITGB3. Cell lysates were subjected to IP with anti-ITCH, anti-ITGB3, or anti-IgG antibody (**A**). After infection with ITCH-overexpressing (ITCH-OE) plasmids in ectopic ESCs, the ITCH expression was measured by Western blot (**B**,**C**) and real-time PCR (**D**); * *p* < 0.05 compared with NC. After overexpressing of ITCH in ectopic ESCs, the ITGB3 expression was measured by Western blot (**E**,**F**); * *p* < 0.05 compared with NC. The effect of ITCH-OE on the ubiquitination of ITGB3 in ectopic ESCs was detected by IP and Western blot (**G**). NC represents negative control groups.

We further confirmed the ITCH-ITGB3 interaction by Cp-IP. As shown in Figure 3A, ITCH co-immunoprecipitated with ITGB3 protein in ectopic ESCs. Reciprocal Co-IP with ITGB3 antibodies also brought down ITCH, confirming a physical interaction between these two proteins. After infection with ITCH-OE plasmids, the expression of ITCH protein significantly increased compared with that of NC (Figure 3B,C), and the expression of ITCH mRNA increased, too (Figure 3D). Furthermore, ITGB3 showed decreased protein expression levels in ITCH-OE ectopic ESCs (Figure 3E,F). Since ITCH is a ubiquitin ligase, we hypothesized that ITCH may regulate the ITGB3 protein level through ubiquitination modification. Interestingly, we found that ITCH-OE significantly increased the ubiquitination of ITGB3 (Figure 3G).

### 3.4. Overexpression of ITGB3 and/or ITCH Regulate the Proliferation of Ectopic ESCs

**Figure 4:**
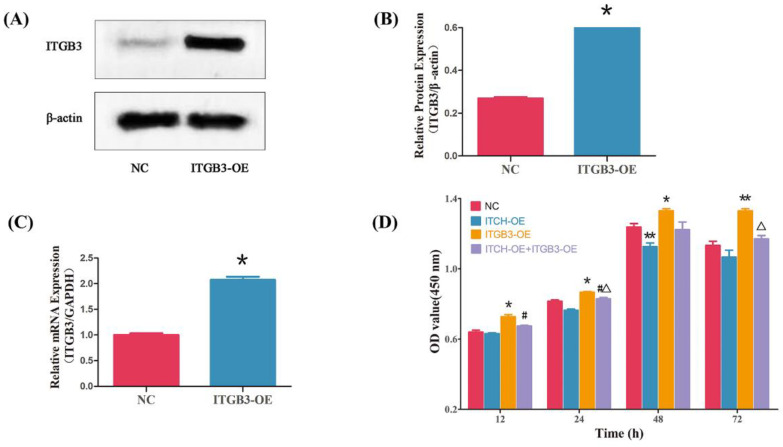
ITGB3 and ITCH regulate the proliferation of ectopic ESCs. After infection with ITGB3-overexpressing (ITGB3-OE) plasmids in ectopic ESCs, the ITGB3 expression was measured by Western blot (**A**,**B**) and real-time PCR (**C**); * *p* < 0.05 compared with NC. Cell growth was evaluated using a CCK8 assay (**D**). Results are presented as mean ± SD of three samples in triplicate, and mean comparisons were performed using two-way ANOVA followed by Tukey’s test. * *p* < 0.05 compared with NC, ** *p* < 0.01 compared with NC, ^#^ *p* < 0.05 compared with ITCH-OE group, and ^Δ^ *p* < 0.05 compared with ITGB3-OE group. NC represents negative control groups.

Our findings prompted us to examine whether ITGB3 and ITCH regulate the proliferation of ectopic ESCs. After infection with ITGB3-OE plasmids, the expression of ITGB3 significantly increased compared with that of NC (Figure 4A–C). CCK8 assays showed that ITGB3-OE significantly promoted cell proliferation at 12, 24, 48, and 72 h; however, after treatment with overexpression of ITCH, the cells’ proliferation ability was reduced at 24 and 72 h. It also showed that ITCH-OE significantly inhibited cell proliferation at 48 h; after treatment with overexpression of ITGB3, the cells’ proliferation ability was increased at 12 and 24 h (Figure 4D). Overall, ITGB3 and ITCH have opposite effects on cell proliferation.

### 3.5. Overexpression of ITGB3 and/or ITCH Regulate the Invasion of Ectopic ESCs

**Figure 5:**
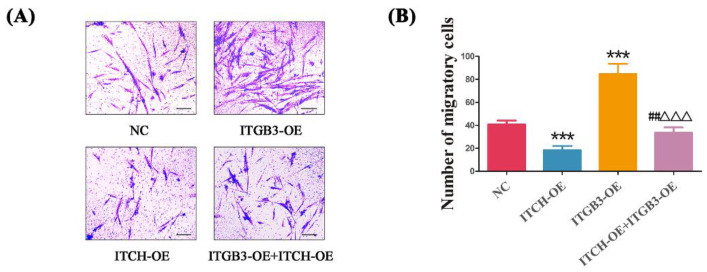
ITGB3 and ITCH regulate the invasion of ectopic ESCs. After infection with ITGB3-OE and/or ITCH-OE plasmids, the migration of ectopic ESCs was detected by transwell migration assay (**A**,**B**), Scale bar: 25 μm. *** *p* < 0.001 compared with NC, ^##^ *p* < 0.01 compared ITCH-OE group, and ^ΔΔΔ^ *p* < 0.001 compared with ITGB3-OE group. Data are expressed as mean ± SD, and data between two groups were compared by two-tailed Student’s *t*-test. NC represents negative control groups.

Given that EM shares similar properties with malignant tumors, including the ability of cell migration, the transwell migration assay was further conducted to evaluate the effect of ITGB3/ITCH on the migration of ectopic ESCs. Cell invasion was measured to examine the effect of ITGB3 and/or ITCH on regulating the development of EM. The number of invading cells in the NC, ITCH-OE, ITGB3-OE, and ITCH-OE + ITGB3OE groups were 40.83 ± 3.31, 18.33 ± 3.72, 84.67 ± 8.89, and 33.50 ± 4.72, respectively (Figure 5A). The transwell migration assays showed that ITGB3-OE significantly enhanced the cells’ invasive ability; however, after treatment with overexpression of ITCH, the cells’ invasive ability was reduced. It also showed that ITCH-OE significantly inhibited the cells’ invasive ability; however, after treatment with overexpression of ITGB3, the cells’ invasive ability was increased (Figure 5B). In general, ITGB3 and ITCH, its ubiquitination protease, have opposite effects on cell invasion.

## 4. Discussion

ITGB3 has been found to play a vital role in tumors. In osteosarcoma, ITGB3 performed the functions of proliferation and cisplatin resistance through the MAPK and VEGF signaling pathways [18]. ITGB3 is upregulated by sponging miR-124-3p, consequently enhancing the proliferation, migration, and invasion of the cells in gastric cancer [19]. ITGB3 also has a central role in intracellular communication via extracellular vesicles, proposed to be critical for cancer metastasis in breast cancer [20]. EM is a gynecological disease with an increasing incidence rate year by year, which severely affects the physical and mental health of women of reproductive age because of its characteristics, such as dysmenorrhea, chronic pelvic pain, and infertility. Although EM is a benign gynecological disease, its biological manifestations are highly similar to malignant tumors, which display features of tumor cells such as angiogenesis, adhesion, growth, invasion, and migration [21]. Human primary ectopic cells are regarded as a better in vitro model system in EM because of their greater homology to the real in vivo situation [22]. In the present study, we detected a notably increased ITGB3 in ectopic ESCs compared with that in normal ESCs, indicating that ITGB3 might be involved in the progression of EM.

Ubiquitylation is a post-translational modification that plays a key role in the degradation of short-life regulatory proteins. Studies have shown that E3 ubiquitin ligases are involved in the origin and development of EM. At present, studies have shown that SMURF1 [17], MDM2 [23], TRIM59 [16], and TRIM65 [24] are upregulated in EM, exhibiting positive cellular biological behavior. However, CHIP [23] and TRIM24 [25] were downregulated in EM, exhibiting opposite effects. Bioinformatics analysis was performed to search for the specific E3 ubiquitin ligase of ITGB3. As a result, ITCH, with a higher confidence level, was found for further study. As a member of the E3 ubiquitin ligases, ITCH plays a vital role in tumorigenesis. When treated under hypoxic conditions, ITCH expression was downregulated in lung cancer (LC) cell lines, which resulted in increased migration and invasion abilities of LC cells [26]. In colorectal carcinoma, the loss of ITCH expression is associated with carcinogenesis, and ITCH might be involved in the Notch-1 pathway during colorectal carcinoma progression [27]. In our research, the expression of ITCH is downregulated in EM; however, it is unclear how ITCH plays a role in EM. Studies have shown that ITCH can block the canonical Wnt pathway and regulate the cell cycle by ubiquitinating phosphorylated disheveled-2 (Dvl2) [28,29]. In melanoma cells, ITCH-mediated BRAF ubiquitination is pivotal in coordinating the signals between cytokines and MAPK pathway activation [30]. This study discovered that the expressions of ITCH and ITGB3 are opposite. To determine their relationship, we further confirmed the ITCH-ITGB3 interaction by Co-IP. Moreover, overexpression of ITCH enhances the ubiquitination of ITGB3 and significantly reduces its expression. We identified how ITCH interacts with ITGB3 and enhanced its expression through ubiquitination in EM.

In ovarian EM, the abnormal invasion promotes the adhesion of ectopic endometrial tissue to the ovary, and its proliferation further leads to the formation of ectopic lesions. In this study, it is reasonable to speculate that ITGB3 and ITCH E3 ubiquitin ligase may be involved in forming EM by regulating their expression. Our data observed that increased ITGB3 significantly promoted cell proliferation and invasion; however, these abilities can be prohibited by overexpression of ITCH. On the contrary, increased ITCH dramatically inhibited cell proliferation and invasion; however, these changes are canceled out for ITGB3 overexpression. These findings indicated that ITGB3 may positively regulate cell biological processes in EM via ubiquitylation of ITCH. Dysregulation of ubiquitin regulation in the ectopic environment may have a crucial role in the pathogenesis of EM. ITGB3 and ITCH E3 ubiquitin ligase interact with each other and exhibit opposite biological behavior in ectopic ESCs. ITGB3 is considered a marker of angiogenesis and is involved in the committed steps of tumor angiogenesis by regulating cell–cell and cell–matrix interactions and its involvement in several signaling pathways [31]. According to the results of Li’s study, the upregulation of ITGB3 activates the PIK3R1/Akt pathway, thereby exerting anti-apoptosis and protective effects on endothelial cells [32]. Wang et al. revealed that silencing the expression of ITGB3 will promote the activation of the MAPK signaling pathway by elevating the phosphorylation of Cx43 and GSK-3β downstream, which inhibits inflammatory reaction and myocardial cell apoptosis, promoting cell proliferation in mice [33]. The possible mechanism by which ITGB3 participates in the formation of EM under the influence of ITCH E3 ubiquitin ligase deserves further investigation. Among the factors contributing to the formation of EM, changes in the immune environment are notable factors. In EM, activating proinflammatory cytokines produced by neutrophils and neutrophil elastase and other related protease mediators secreted by neutrophils will increase the risk of angiogenesis and tissue invasion of patients with EM [34]. Luo et al. discovered that ITGB3 affects tumor immunity by acting on the innate immune system, which showed transcriptional upregulation, and through a progressive increase of surface expression after neutrophil infiltration [35]. Further research is needed to determine whether the interaction between ITGB and ITCH E3 ubiquitin ligase promotes the formation of EM by altering the immune environment.

## 5. Conclusions

This study demonstrated, for the first time, that ITGB3 interacts with and ubiquitinates ITCH. Upregulation of ITGB3 induces the proliferation and migration of ectopic ESCs. The limitations of this study have to be acknowledged. Due to ethical factors, our study lacks the eutopic endometrium as a control group. Additionally, an in vivo study will significantly increase the impact of the findings. In addition, further research is needed for the underlying molecular mechanism of how ITGB3 positively regulates the biological behavior of the cells and the detailed mechanism by which ITCH can interact with ITGB3. This research may unravel one of the essential mechanisms of EM, providing novel treatment strategies for EM.

## Figures and Tables

**Table 1:** Antibody information.

Antibodies	Working Concentration	Manufacturers
***CK19***	1:200	ZSGB-BIO (TA336915)
***Vimentin***	1:150	ZSGB-BIO (TA801250)
***ITGB3***	1:1000	Proteintech (66952-1-Ig)
***ITCH***	1:800	Proteintech (67757-1-Ig)
***Ub***	1:600	Proteintech (10201-2-AP)
***β-actin***	1:3000	Servicebio (GB15003)

**Table 2:** Primer sequences used in this study.

Gene	Sequences
***ITCH***	F: 5′-GACCGGCTGCCATCTTAGTC-3′
	R: 5′-GGGTTAAGGCGTTGTCTCCA-3′
***ITGB3***	F: 5′-GACTTGGGCAGGGTACAGAC-3′
	R: 5′-AGACCTTCAAGACTGGCTGC-3′
***GAPDH***	F: 5′-GACAGTCAGCCGCATCTTCT-3′
	R: 5′-GCGCCCAATACGACCAAATC-3′

## Data Availability

The data of this study are available from the corresponding author upon reasonable request.
